# Effect of *Boswellia serrata* on Pain Intensity, Central and Peripheral Sensitization, and Pain Modulation in Healthy Volunteers—A Randomized, Double-Blind, Placebo-Controlled, Cross-Over Pilot Trial

**DOI:** 10.3390/nu18121839

**Published:** 2026-06-06

**Authors:** Sascha Hammer, Marco Reiser, Mathias Bader, Jakob Pannold, Angelika Moser, Maximilian Niederer, Anselm Johannes Schlemmer, Sebastian Labenbacher, Kordula Lang-Illeviech, Helmar Bornemann-Cimenti

**Affiliations:** 1 Department of Anesthesiology and Intensive Care Medicine 1, Medical University of Graz, 8010 Graz, Austria; sascha.hammer@medunigraz.at (S.H.); marco.reiser@stud.medunigraz.at (M.R.); mathias.bader@medunigraz.at (M.B.); angelika.moser@stud.medunigraz.at (A.M.); maximilian.niederer@medunigraz.at (M.N.); sebastian.labenbacher@medunigraz.at (S.L.); 2 Department of Anesthesiology and Intensive Care Medicine 2, Medical University of Graz, 8010 Graz, Austria; jakob.pannold@medunigraz.at; 3 Department of General Radiology, Medical University of Graz, 8010 Graz, Austria; anselm.schlemmer@medunigraz.at; 4 Department of Anesthesiology and Intensive Care Medicine, LKH Güssing, 7540 Güssing, Austria; kordula.lang-illievich@medunigraz.at

**Keywords:** *Boswellia serrata*, central sensitization, conditioned pain modulation, capsaicin model, hyperalgesia, quantitative sensory testing

## Abstract

Background: *Boswellia serrata* has traditionally been used in Ayurvedic medicine for its anti-inflammatory and antioxidant properties. Although several studies support clinical analgesic efficacy, the underlying mechanisms have not been investigated in human experimental pain models. This randomized, double-blind, placebo-controlled, crossover pilot trial aimed to examine the mode of action of *Boswellia serrata* to differentiate between its peripheral and central effects. This exploratory pilot study was designed to generate preliminary effect size estimates and assess functional pain-processing outcomes, rather than to provide definitive evidence of clinical efficacy. Methods: Twelve healthy volunteers were recruited and received either 300 mg of *Boswellia serrata* extract or a visually identical placebo twice daily for 28 days, separated by a 4-week washout period. Pain and sensitization were induced using a topical capsaicin model. Outcomes included spontaneous pain intensity, mechanical allodynia, pinprick hyperalgesia, thermal thresholds, and conditioned pain modulation, alongside psychological assessments of mood, anxiety, sleep, and structured adverse-event monitoring. Results: Results showed no significant difference in the primary endpoint of spontaneous pain intensity between Boswellia and placebo (VAS 43 ± 21 vs. 47 ± 17; d = 0.18; *p* = 0.539). Conclusions: While *Boswellia serrata* did not significantly reduce acute peak pain in this model, the observed trends suggest a potential multi-level modulatory influence on nociceptive processing and endogenous pain inhibition. These findings warrant larger clinical trials to further elucidate its therapeutic potential, particularly in populations with impaired pain modulation.

## 1. Introduction

*Boswellia serrata* (family *Burseraceae*) is an oleogum resin-producing tree native to the Indian subcontinent whose dried exudate has been employed in Ayurvedic medicine for centuries as an antioxidant and anti-inflammatory remedy in conditions ranging from rheumatoid arthritis and asthma to chronic inflammatory bowel disease [1,2]. The principal bioactive constituents are the β-configured pentacyclic triterpenic acids—most notably 3-acetyl-11-keto-β-boswellic acid (AKBA), 11-keto-β-boswellic acid (KBBA), β-boswellic acid (BBA), and 3-acetyl-β-boswellic acid (ABBA)—which together account for approximately 14% of the lipophilic extract fraction, with the β-configured derivatives exhibiting substantially greater pharmacological potency than their α-isomers [3,4]. Available as an over-the-counter dietary supplement in Austria and across much of Europe, *B. serrata* has attracted growing scientific interest as a potentially well-tolerated alternative to conventional non-steroidal anti-inflammatory drugs.

The clinical evidence for its analgesic efficacy is most robust in chronic inflammatory pain conditions. Randomized controlled trials and subsequent meta-analyses have demonstrated significant reductions in pain intensity and functional impairment in osteoarthritis of the knee, with benefits reported across a range of standardized extract doses [5]. Positive results have further been reported in spondyloarthritis, asthma, and inflammatory bowel disease [1,2].

The pharmacological mechanisms underlying these clinical effects are, however, only partially elucidated and contested. The long-canonical account attributed the principal activity to AKBA-mediated selective inhibition of 5-lipoxygenase (5-LO) and consequent suppression of pro-inflammatory leukotriene synthesis, as well as to interference with nuclear factor kappa B (NF-κB) signaling and inhibitor of kappa B (IκB) kinases [4]. This account was substantially complicated by pharmacokinetic data demonstrating that plasma concentrations of AKBA following oral administration fall far below the threshold required for 5-LO inhibition in vitro, whereas β-boswellic acid, present at roughly 100-fold higher plasma concentrations, inhibits microsomal prostaglandin E_2_ synthase-1 (mPGES-1) and the serine protease cathepsin G, identifying these as the more plausible molecular targets in the human setting [6]. Anti-inflammatory activity has additionally been documented in carrageenan-induced animal models of paw oedema [7], and preclinical data suggest further neuromodulatory properties, including modulation of serotonergic pathways and acetylcholinesterase inhibition [8].

However, it remains unresolved whether the analgesic effects of *Boswellia serrata* in humans are mediated primarily at the peripheral level, through suppression of neurogenic inflammation at sites of tissue injury, or whether they also involve central nociceptive processing, including modulation of spinal sensitization and descending inhibitory pathways. This distinction carries direct therapeutic implications: a predominantly peripheral mode of action would favor its application in acute somatic and inflammatory pain, whereas a demonstrable central component would support its use in conditions characterized by central sensitization, impaired conditioned pain modulation, or neuropathic features [9,10,11,12,13]. To date, no human experimental study has systematically addressed this question. The aim of the present study was therefore to examine the mode of action of *Boswellia serrata* in a validated experimental human pain model, specifically designed to enable mechanistic differentiation between peripheral and central analgesic effects.

## 2. Materials and Methods

**Figure 1:**
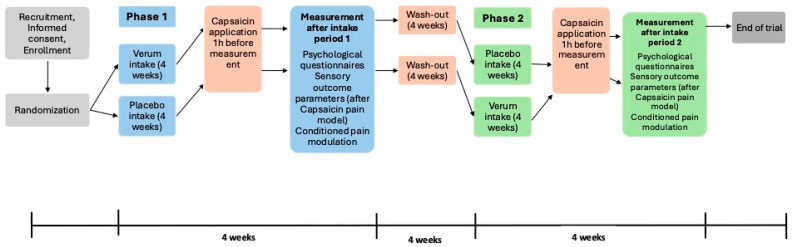
Timeline of the trial.

This study was designed as a randomized, double-blind, placebo-controlled, crossover pilot trial at the Department of Anesthesiology and Intensive Care Medicine, Medical University of Graz. The type of pain investigated in this study was experimentally induced acute nociceptive pain, generated by topical capsaicin application and characterized by neurogenic inflammation, spontaneous burning pain, primary sensitization at the application site, and secondary hyperalgesia/allodynia in the surrounding area. The total duration of study participation for each subject was 12 weeks, comprising two 28-day treatment phases separated by a 4-week wash-out period (Figure 1). Twelve healthy volunteers were recruited by public notices at the Medical University of Graz, the University of Graz, and the Graz University of Technology. Using computer-generated randomization (https://www.atom.com/name/Randomizer, accessed on 27 September 2025), participants were assigned in a 1:1 ratio to one of two treatment sequences (active treatment/placebo vs. placebo/active treatment). Group assignment was conducted under strict confidentiality by a person not otherwise involved in outcome assessment or statistical analysis. Participants, study staff involved in assessments, and statistical analysts were blinded to treatment allocation. Randomization was performed despite the pilot nature of the study to minimize allocation bias, reduce potential sequence effects, and assess the feasibility of a randomized crossover design for future confirmatory trials.

### 2.1. Registration

The study was preregistered at Clinicaltrials.gov on 31 July 2025 (ID: NCT07109843).

### 2.2. Inclusion and Exclusion Criteria

Eligible participants were adults aged 18 years or older who could provide written informed consent. Exclusion criteria encompassed pregnancy or breastfeeding; renal or hepatic insufficiency; pre-existing neurological, dermatological, or cardiovascular disease; chronic pain disorders or regular analgesic use; and concomitant treatment with anticoagulants, antidepressants, monoamine oxidase inhibitors, CYP-modifying agents, or St. John’s Wort. Participants with known hypersensitivity to *Boswellia serrata* or capsaicin were likewise excluded.

### 2.3. Interventions

Participants received 56 capsules containing either *Boswellia serrata* extract (Boswellin^®^ Super, 300 mg per capsule, Sabinsa Europe GmbH, Langen, Germany) or a visually identical placebo (maltodextrin-filled capsules prepared by Casa Medica Pharmacy Dr. Peyer, Graz, Austria). According to the manufacturer’s specification, the extract contained 75% total boswellic acids, including at least 50% β-boswellic acid and 30% 3-acetyl-11-keto-β-boswellic acid. Capsules and packings were indistinguishable in size, shape, and appearance. The treatment regimen consisted of 300 mg twice daily for 28 days, corresponding to a total daily dose of 600 mg. After a 4-week washout period, participants crossed over to the alternative treatment for another 28 days. Capsules were taken in the morning and evening, with electronic reminders sent via mobile phone to maximize adherence. The selected dose of 600 mg/day was based on the manufacturer’s recommended intake for Boswellin^®^ Super and on previous clinical studies using standardized *Boswellia serrata* extracts in inflammatory pain conditions [14]. Blinding was maintained by coded containers labelled “Substance 1” or “Substance 2.” Based on Kulkarni et al. [15], the longest reported elimination half-life of *Boswellia* constituents (AKBA) is approximately 6.8 ± 3.0 h. Complete drug elimination is generally assumed after at least five half-lives, corresponding to approximately 34–50 h for AKBA. Thus, the 4-week washout period greatly exceeded the estimated time required for systemic clearance and was considered sufficient to minimize potential carryover effects between study phases. At the end of each 4-week intake period, either active treatment or placebo, participants underwent the experimental pain testing procedure. Thus, each participant completed two testing sessions, one after each treatment condition.

### 2.4. Experimental Pain Induction

To assess spontaneous pain and sensitization, the topical capsaicin model was used. The type of pain investigated in this study was experimentally induced acute nociceptive pain, generated by topical capsaicin application and characterized by neurogenic inflammation, spontaneous burning pain, primary sensitization at the application site, and secondary hyperalgesia/allodynia in the surrounding area. A 30 × 30 mm patch containing 5.76 mg capsaicin (640 µg/cm^2^, Qutenza, Grünenthal, Vienna Austria) was applied to the volar forearm for 60 min. Skin temperature was checked prior to application with an infrared thermometer and normalized (>30 °C) with a warming blanket if necessary. After removal, the site was cleaned with neutral gel, according to the manufacturer’s instructions. This model induces neurogenic inflammation by activating transient receptor potential vanilloid 1 (TRPV1) receptors, producing erythema, oedema, spontaneous pain, and both primary (peripheral) and secondary (central) hyperalgesia [16]. Measurements were conducted immediately after patch removal, when sensitization peaks. At the second measurement, the other forearm was used to avoid applying the capsaicin patch to the same site.

### 2.5. Outcome Measures

Outcome assessment was performed according to a predefined hierarchy of clinical and experimental endpoints. The primary endpoint was spontaneous pain intensity after capsaicin patch removal, assessed using a visual analogue scale (VAS). Secondary endpoints included quantitative sensory testing (QST)-based measures of peripheral and central sensitization, conditioned pain modulation (CPM), cold pain tolerance (CPT), psychological outcomes, adherence, and safety. Outcome assessment was performed in accordance with recommendations of the German Research Network on Neuropathic Pain (DFNS) and previously published experimental pain studies. QST and CPM were used as validated functional measures to assess peripheral sensitization, central sensitization, and descending endogenous pain modulation [17,18,19,20]. Adherence to active treatment and placebo intake was assessed by counting the number of capsules remaining at Measurement 1 and Measurement 2. Adherence was calculated as the proportion of capsules consumed relative to the number dispensed. Participants with no remaining capsules at the respective time point were considered to have 100% adherence. In this model, peripheral sensitization was operationalized as altered thermal sensitivity within the primary capsaicin-treated area, assessed by heat detection threshold and heat pain threshold. Central sensitization was operationalized as secondary-zone mechanical hyperalgesia, mechanical allodynia, and temporal summation/wind-up ratio, while CPM was used as a functional measure of descending endogenous pain inhibition.

### 2.6. Primary Endpoint

The primary endpoint was spontaneous pain intensity immediately after capsaicin patch removal. Pain intensity was assessed using a visual analogue scale (VAS, 0–100), with 0 indicating no pain and 100 indicating the worst imaginable pain. This endpoint was chosen to quantify acute capsaicin-induced spontaneous nociceptive pain.

### 2.7. Secondary Endpoints

Secondary endpoints were selected to characterize distinct components of capsaicin-induced pain processing, including peripheral sensitization, secondary central sensitization, temporal summation, descending endogenous pain modulation, cold pain tolerance, and psychological factors potentially influencing pain perception.

Mechanical allodynia: We measured the distance of the unpleasant sensation evoked using von Frey filaments (128 mN) applied at 5 mm intervals along four radial tracks from the area where the capsaicin patch was placed. For each track, we performed one measurement moving toward the center and one moving away. These eight distances were then averaged [21].

Pinprick hyperalgesia and wind-up phenomena: To evaluate temporal summation, the perceived intensity of a single pinprick stimulus (256 mN) was compared to a train of 10 identical stimuli delivered at 1 Hz. This sequence was applied to a 1 cm^2^ area, approximately 5 cm proximal to the center of the capsaicin patch. Participants rated the pain of the initial stimulus and the final stimulus of the train using a 1–100 numeric rating scale (NRS). A wind-up ratio (WUR) was then calculated by dividing the pain rating of the tenth stimulus by that of the first [22].

Thermal thresholds: Heat detection and heat pain thresholds were measured using the TSA-II NeuroSensory Analyzer (Medoc Ltd., Ramat Yishai, Israel). A thermode was positioned on the capsaicin patch area, and participants indicated the onset of both the first thermal sensation (detection threshold) and the first painful sensation (pain threshold) by pressing a stop button. Each measurement was performed in triplicate, and the values were averaged to determine the mean threshold [18].

Conditioned pain modulation (CPM) and cold pain tolerance (CPT): CPM was assessed using the “pain inhibits pain” paradigm to evaluate endogenous analgesia [23]. The test stimulus—pressure pain threshold (PPT)—was measured at the M. pollicis brevis of the non-dominant hand using a pressure algometer (1 cm^2^ probe). PPT was determined by gradually increasing pressure until the first report of pain [17]. For the conditioning stimulus, participants immersed their dominant hand and wrist in cold water until a pain intensity of 40 on a 0–100 NRS was reached; the duration of immersion served as the measure for cold pain tolerance (CPT). Immediately following hand withdrawal, the PPT was reassessed at the original site. The CPM response was defined as the change in pressure pain threshold from baseline.

Psychological assessment: As part of the psychological assessment, depressive symptoms were assessed using the Beck Depression Inventory (BDI-II), and anxiety was measured using the Beck Anxiety Inventory (BAI) [24,25]. Sleep quality was assessed using the Pittsburgh Sleep Quality Index (PSQI) [26]. In addition, well-being was evaluated using the WHO-5 Well-Being Index [27].

### 2.8. Standardization of Clinical Evaluation Criteria

To minimize confounding and improve comparability between treatment conditions, all assessments were performed according to the same standardized procedure in both study periods. Measurements were conducted immediately after capsaicin patch removal, when sensitization was expected to peak. In the second measurement session, the contralateral forearm was used to avoid local carryover effects from repeated capsaicin application at the same site. The randomized crossover design allowed each participant to serve as their own control, thereby reducing interindividual variability in pain sensitivity. Treatment-related effects were evaluated as within-subject differences between Boswellia serrata and placebo. Potential confounding was further reduced by the 4-week washout period, blinded outcome assessment, standardized capsaicin dose and application time, standardized skin temperature control before capsaicin application, and predefined exclusion criteria for chronic pain, regular analgesic use, relevant comorbidities, and potentially interacting co-medications.

### 2.9. Sample Size Calculation

As no prior data were available, an effect size estimation was performed. Assuming α = 0.05, β = 0.20 (power 0.8), a paired *t*-test design indicated that 10 participants would be sufficient to detect an effect size ≥ 1. Anticipating a dropout rate of 10–20%, a total of 12 participants were recruited. This aligns with a previously published study of similar design by Lang-Illevich et al. [18].

### 2.10. Statistical Analysis

Data were collected from standardized case report forms and entered into a study database. Analyses were performed in R (R Core Team, Version 4.5.1). Continuous data are summarized as mean ± SD per condition to describe variability and as mean within-subject difference with a 95% confidence interval to indicate the inferential precision of effect estimates, in accordance with the established convention of separating descriptive variability measures from inferential precision measures [28,29,30]. Categorical data are reported as n (%). Given the crossover design, the pre-specified primary analysis compared spontaneous capsaicin-induced pain intensity, measured using the visual analogue scale (VAS), between Boswellia serrata and placebo using a paired *t*-test on within-subject differences. Cohen’s dz was reported as the corresponding effect size [31]. Secondary endpoints were analyzed analogously and, given the exploratory nature of this pilot trial, were not adjusted for multiplicity. The assumption of approximate normality of the within-subject differences was assessed for each endpoint using the Shapiro–Wilk test (Supplementary Table S1). Where this assumption was not supported, results were corroborated using the Wilcoxon signed-rank test as a non-parametric sensitivity analysis (Supplementary Table S1). To facilitate sample-size planning for confirmatory trials, three Cohen’s d variants, dz, dav, and drm, are reported in Supplementary Table S1, following Lakens [31]. The two-sided significance threshold was set at *p* < 0.05.

### 2.11. Safety and Risk Assessment

Safety was assessed throughout the study by monitoring adverse events related to both the oral study intervention and the capsaicin pain model. At each study visit and before each experimental pain testing session, participants were asked about adverse symptoms, changes in health status, and any medication use since the previous contact. Particular attention was paid to known or plausible adverse effects of *Boswellia serrata*, including gastrointestinal symptoms such as abdominal discomfort, nausea, diarrhea, allergic reactions, and symptoms suggestive of drug interactions. Local tolerability of the capsaicin patch was monitored during and after application, including burning pain, erythema, itching, or excessive discomfort. Severe pain, defined as pain intensity greater than NRS 6 during capsaicin application, would have led to immediate patch removal and supportive care. Adverse events were defined as any unfavorable medical occurrence during the study period, irrespective of suspected causality. Events were planned to be categorized according to severity, timing, relationship to the study intervention, and need for treatment or discontinuation. Serious adverse events were defined as events resulting in hospitalization, persistent disability, life-threatening conditions, or death. Participants using anticoagulants, antidepressants, monoamine oxidase inhibitors, CYP-modifying agents, or St. John’s Wort were excluded to minimize potential interaction risks, as clinically relevant interactions of *Boswellia serrata* remain incompletely characterized. *Boswellia serrata* is marketed as a dietary supplement under EU Directive 2002/46/EC and is generally considered safe; reported mild adverse effects include abdominal pain, diarrhea, nausea, and rare allergic reactions [32,33]. No serious adverse events were expected.

## 3. Results

Between October and December 2025, 12 healthy volunteers participated in the pilot study. The participants were placed in two groups, equal in size and sex—two female and four male—as well as consisting of similar mean ages. All participants finished the study as per protocol. Table 1 summarizes the descriptive characteristics of this trial. To improve transparency of the crossover design, phase-specific descriptive values after phase 1 and after phase 2 are provided in Supplementary Table S2.

### 3.1. Spontaneous Pain

The primary endpoint, spontaneous pain intensity following capsaicin patch removal, did not differ significantly between conditions. Mean VAS scores were 43.00 ± 21.02 under *Boswellia serrata* and 46.67 ± 16.59 under placebo, corresponding to a small effect size (Cohen’s d = 0.183; *p* = 0.539), as shown in Table 2.

### 3.2. Peripheral Sensitization (HPT, HDT)

Peripheral sensitization was evaluated using thermal thresholds measured at the capsaicin-treated area, whereas central sensitization was evaluated using secondary mechanical hyperalgesia, mechanical allodynia, and wind-up ratio outside the primary stimulation area. CPM was analyzed separately as an index of endogenous descending pain modulation. Regarding the HPT, a trend toward decreased thresholds was observed under Boswellia treatment with temperature at 34.77 ± 0.64 °C under *Boswellia serrata* vs. 37.59 ± 2.36 °C under placebo, yielding a small effect size, which did not reach significance (d = 0.218, *p* = 0.466). The HDT remained essentially unchanged, with the temperature at 34.77 ± 0.64 °C in the Boswellia group and at 34.79 ± 0.74 °C in the placebo group (d = 0.028, *p* = 0.924).

### 3.3. Central Sensitization (Hyperalgesia, WUR, Allodynia)

Boswellia intake resulted in reduced pain ratings for pinprick stimuli in the secondary zone, with values at 20.17 ± 12.66 in the Boswellia group vs. 22.83 ± 15.22 in the placebo group, demonstrating a small-to-medium effect size (d = 0.303, *p* = 0.317). In contrast, the spatial extent of allodynia, with 35.26 ± 9.56 in Boswellia vs. 34.53 ± 8.22 in placebo (d = 0.054, *p* = 0.844), and temporal summation, with values of 35.42 ± 17.14 in Boswellia and 34.50 ± 13.73 in placebo (d = 0.042, *p* = 0.887), showed negligible changes.

### 3.4. Conditioned Pain Modulation (CPM) and Cold Pain Tolerance (CPT)

CPM showed a medium effect size, with values of 107.21 ± 19.46 under *Boswellia serrata* and 113.73 ± 13.81 under placebo; however, this difference did not reach statistical significance (d = 0.505, *p* = 0.108). The cold pain tolerance, however, remained stable with values at 18.13 ± 8.61 for *Boswellia serrata* vs. 18.99 ± 9.36 for placebo (d = 0.387, *p* = 0.808).

### 3.5. Psychological Outcomes and Quality of Life

A medium effect size (d = 0.475, *p* = 0.128) was observed for the reduction in anxiety symptoms (3.58 ± 5.02 vs. 4.92 ± 6.86). Well-being scores showed an improvement trend with a medium effect size (17.67 ± 3.03 vs. 16.67 ± 3.92 (d = 0.415, *p* = 0.179)). A small effect on sleep quality (3.67 ± 2.02 vs. 4.33 ± 1.87 (d = 0.339, *p* = 0.266)) was noted. On the depression score BDI-II, with values at 4.33 ± 5.02 vs. 4.08 ± 5.16 (d = 0.064, *p* = 0.830), an absence of an effect was observed.

### 3.6. Adverse Event

No adverse events, serious adverse events, treatment discontinuations, or clinically relevant local reactions to capsaicin were reported during *Boswellia serrata* or placebo treatment.

## 4. Discussion

In this pilot study, *Boswellia serrata* did not significantly reduce acute capsaicin-induced spontaneous pain. Because none of the observed differences reached statistical significance, the findings should be interpreted as exploratory and hypothesis-generating. The observed effect sizes may be useful for planning future studies, but they do not establish clinical efficacy or confirm a specific mechanism of action. Therefore, the present findings should not be interpreted as evidence of an analgesic effect, but rather as preliminary functional data intended to inform the design, dosing, and sample size of future confirmatory trials.

Some QST and CPM outcomes showed small-to-medium effect sizes, particularly secondary mechanical hyperalgesia and CPM. However, these effects were non-significant and should not be equated with clinically relevant treatment effects, especially because the study included healthy volunteers and used an experimental acute pain model rather than a clinical pain population.

Importantly, the present study should be interpreted as an investigation of the functional mode of action of *Boswellia serrata* rather than as a direct molecular mechanistic study. The capsaicin model allowed the evaluation of experimentally induced acute nociceptive pain, including spontaneous pain, primary peripheral sensitization, secondary central sensitization, and descending endogenous pain modulation. Accordingly, QST parameters and CPM were used as validated functional surrogate markers to differentiate peripheral and central components of nociceptive processing.

The absence of a significant effect on spontaneous pain stands in apparent contrast to the analgesic efficacy of *Boswellia serrata* reported in chronic inflammatory conditions, such as osteoarthritis and spondyloarthritis [9,34]. A plausible explanation for this phenomenon might be in the nature of the experimental paradigm itself. High-dose topical capsaicin (640 µg/cm^2^ for 60 min) induces intense, phasic activation of TRPV1 receptors on C-fibers, generating a nociceptive barrage of a magnitude that likely exceeds the modulatory capacity of boswellic acids at the dose administered [35]. Under conditions of such an acute and supramaximal stimulus, a ceiling effect on peak pain intensity is recognized, rendering pharmacological interventions with primarily anti-inflammatory or neuromodulatory properties difficult to detect [36,37].

One key observation was the dissociation between thermal detection and pain thresholds, which might suggest a selective interaction with the somatosensory system. While the heat detection threshold (HDT) remained unchanged, a slight, non-significant decrease in the heat pain threshold (HPT) was observed under *Boswellia serrata*. This finding may suggest a potential trend toward increased heat sensitivity, with participants reporting that heat can be uncomfortable at lower temperatures. It seems that the preservation of HDT, along with the modulation of HPT, could be consistent with selective effects on nociceptive fibers, such as C and Aδ fibers [35]. It may be possible that this could result in the sparing of Aβ fiber-mediated detection pathways [38,39]. While the HPT change direction was pro-nociceptive in this group, the dissociation itself lends support to the hypothesis that boswellic acids, particularly acetyl-11-keto-β-boswellic acid, may interact with pathways such as 5-lipoxygenase and TRPV1 to modulate neurogenic inflammation [8].

Regarding central sensitization, a trend toward a reduction in pinprick hyperalgesia ratings in the secondary zone (d = 0.302) could be indicative of a potential dampening of the heterosynaptic long-term potentiation of dorsal horn neurons [40]. However, the spatial area of allodynia remained stable. This could suggest that *Boswellia serrata* may modulate the intensity of the sensory response within the sensitized zone rather than shrinking the physical area of central plasticity [41]. It appears that the observed lack of effect on the wind-up ratio (d = 0.042) might suggest that frequency-dependent summation mechanisms in the spinal cord are not the primary target of Boswellia at the chosen dosage. However, these findings should be interpreted as functional evidence of altered central pain processing and not as direct proof of molecular changes within the central nervous system.

The largest observed effect was found for CPM, which had a moderate effect size (d = 0.50). While the findings may not be statistically significant, they could offer valuable insights from a mechanistic perspective. CPM appears to reflect the efficiency of descending inhibitory pathways, and impairments in this system may be associated with an increased risk of pain chronification [42,43]. The observed difference in CPM showed a medium effect size but did not reach statistical significance and should therefore be interpreted cautiously. These findings may indicate that CPM is a relevant endpoint for future studies, but they do not provide evidence that *Boswellia serrata* enhances endogenous pain inhibition [23,44]. This suggests that the pharmacological profile of Boswellia might extend beyond peripheral anti-inflammatory effects, hinting at the possibility of a central modulatory component. CPT remained largely unchanged, which again could reinforce the hypothesis of a possible impact on central modulation.

The secondary outcomes should also be interpreted in the context of their established score ranges. For anxiety (BAI, range 0–63) and general well-being (WHO-5, range 0–25), the observed moderate effect size might be in alignment with preclinical and pharmacological evidence suggesting neurochemical modulatory properties of boswellic acids, including effects on serotonergic and GABAergic pathways [8,45,46]. Still, the mean scores for both groups remain well within the “minimal anxiety” (0–7) and “high well-being” (>13) categories, respectively. The absence of a comparable effect on depressive symptoms (BDI-II, range 0–63) and sleep quality (PSQI, range 0–21) may be plausibly attributable to a floor effect. With baseline BDI-II scores (minimal range 0–13) and PSQI scores (good sleep threshold < 5), there was negligible room for measurable improvement in this healthy population. This phenomenon is well documented in intervention studies enrolling participants without clinically meaningful symptom burden, where the lack of baseline distress limits the ability to detect pharmacological modulation [47].

The objective of this study was to explore the distinction between peripheral and central mechanisms of *Boswellia serrata* within a controlled human experimental pain model. As one of the first studies to combine QST and CPM outcomes in this context, it provides initial effect size estimates that may inform the design and power calculations of future trials. The findings provide preliminary effect size estimates for future studies but do not establish a multi-level analgesic profile of *Boswellia serrata*. Larger studies with adequate power, optimized dosing, and biochemical verification are required before conclusions regarding peripheral or central mechanisms can be drawn.

### 4.1. Limitations

The most important methodological limitation concerns statistical power. In the absence of prior effect size estimates for *Boswellia serrata* in experimental human pain models, the sample size calculation was based on the assumption of a large effect (Cohen’s d ≥ 1.0), yielding a minimum sample of n = 10 (α = 0.05, power = 0.80). The effect sizes observed ranged from small to medium (d = 0.18–0.51); for the largest observed effect—conditioned pain modulation (d = 0.505)—an adequately powered study would have required approximately 34 participants, rising to roughly 90 for the hyperalgesia endpoint (d = 0.302). The present study was therefore substantially underpowered to detect effects of the magnitude observed. It follows that the absence of statistical significance cannot be interpreted as evidence of no effect. Therefore, the absence of statistically significant findings should be interpreted in the context of limited power, and the observed effect sizes should primarily be regarded as estimates for planning future confirmatory trials. The primary contribution of this pilot study is, accordingly, the generation of empirical effect size estimates to inform the power calculations of adequately sized confirmatory trials. A further limitation concerns the administered dose. The total daily dose of 600 mg (2 × 300 mg of Boswellin^®^ Super) lies at the lower boundary of the range employed in published clinical trials, in which effective doses have spanned from 300 mg to 6 g per day depending on the degree of standardization and the clinical indication [14,34]. The comparatively low doses used in those studies that employed the same standardized Boswellin^®^ Super extract lend some support to the adequacy of the chosen regimen [5]; moreover, the dose selected is consistent with the manufacturer’s recommended intake. However, boswellic acids, particularly AKBA, have limited oral bioavailability, and the selected dose may have been insufficient to induce measurable systemic or central effects in this experimental pain model. Future studies should therefore consider higher doses, bioavailability-enhanced formulations, and pharmacokinetic monitoring [5]. Future studies should therefore consider higher doses, bioavailability-enhanced formulations, or pharmacokinetic monitoring to establish a reliable exposure–response relationship in experimental pain models. Another limitation is the absence of molecular biomarker assessment. Central and peripheral sensitization were evaluated using validated functional QST-based measures rather than molecular markers. Future studies should include inflammatory and neurochemical biomarkers to further clarify the biological mechanisms underlying the observed effects. A further limitation is the absence of independent batch-specific chemical analysis of the investigated extract. The composition was based on the manufacturer’s specification for Boswellin^®^ Super, but no additional HPLC or mass spectrometric verification was performed within the present pilot study. Future trials should include batch-specific phytochemical characterization to improve reproducibility and allow for more precise exposure–response interpretation.

### 4.2. Implications for Future Research

Based on the findings of this study, several improvements for prospective research emerge to further delineate the therapeutic potential of *Boswellia serrata*. Future and adequately powered clinical trials could consist of participants with chronic inflammatory pain syndromes, particularly those with a documented dysfunction in CPM. To strengthen the mechanistic understanding of the results, the inclusion of objective biomarkers such as C-reactive protein (CRP) and pro-inflammatory cytokines like interleukin 6 (IL-6) can be recommended. Furthermore, the incorporation of plasma level monitoring of 3-acetyl-11-keto-β-boswellic acid (AKBA) should be considered to ensure therapeutic concentrations are reached, as well as using higher dosages or bioavailability-optimized formulations to maximize clinical efficacy. While the capsaicin model used in this study provided valuable initial insight, transitioning from a phasic to a tonic pain model would more accurately reflect the nature of chronic pain.

## 5. Conclusions

In this randomized crossover pilot study, *Boswellia serrata* did not significantly reduce acute capsaicin-induced spontaneous pain or peripheral thermal sensitization. Therefore, the study does not provide definitive evidence for a peripheral analgesic effect in this experimental acute nociceptive pain model. Exploratory differences in secondary mechanical hyperalgesia and CPM were observed but did not reach statistical significance. These findings should therefore be interpreted as hypothesis-generating and primarily useful for planning larger, adequately powered studies. Future trials should include optimized dosing, pharmacokinetic monitoring, and batch-specific phytochemical characterization to further clarify the potential role of *Boswellia serrata* in pain modulation.

## Figures and Tables

**Table 1:** Participant characteristics.

	Group 1	Group 2
Sex (f/m)	2/4	2/4
Age (years)	32.8 ± 4.5	29.2 ± 2.3
Height (cm)	177.0 ± 7.6	175.2 ± 9.1
Weight (kg)	68.5 ± 9.9	72.0 ± 5.6
Adherence to *Boswellia serrata* intake	100%	100%
Adherence to Placebo intake	100%	100%

**Table 2:** Results of the trial. VAS: visual analogue scale; HPT: heat pain threshold; HDT: heat detection threshold; WUR: wind-up ratio; CPM: conditioned pain modulation; CPT: cold pain tolerance; BAI: Beck Anxiety Inventory; PSQI: Pittsburgh Sleep Quality Index; BDI: Beck Depression Inventory. The newly added Mean diff [95% CI] column reports the mean within-subject difference (Boswellia minus placebo) with its 95% confidence interval as the inferential precision measure.

Parameter	* Boswellia serrata *	Placebo	Mean Diff [95% CI]	Effect Size (d)	*p*-Values
BAI	3.58 ± 5.02	4.92 ± 6.86	−1.33 [−3.12; 0.45]	0.475	0.128
BDI	4.33 ± 5.02	4.08 ± 5.16	+0.25 [−2.25; 2.75]	0.064	0.830
CPM (%)	107.21 ± 19.46	113.73 ± 13.81	−6.52 [−14.74; 1.69]	0.505	0.108
CPT (s)	18.13 ± 8.61	18.99 ± 9.36	−0.87 [−8.52; 6.78]	0.387	0.808
Distance of allodynia (mm)	35.26 ± 9.56	34.53 ± 8.22	+0.73 [−7.88; 9.34]	0.054	0.844
HDT (°C)	34.77 ± 0.64	34.79 ± 0.74	−0.02 [−0.62; 0.57]	0.028	0.924
HPT (°C)	36.84 ± 2.17	37.59 ± 2.36	−0.75 [−2.92; 1.43]	0.218	0.466
Hyperalgesia (0–100)	20.17 ± 12.66	22.83 ± 15.22	−2.67 [−8.27; 2.93]	0.303	0.317
PSQI	3.67 ± 2.02	4.33 ± 1.87	−0.67 [−1.92; 0.59]	0.339	0.266
VAS (0–100)	43.00 ± 21.02	46.67 ± 16.59	−3.67 [−16.38; 9.05]	0.183	0.539
WHO-5	17.67 ± 3.03	16.67 ± 3.92	+1.00 [−0.53; 2.53]	0.415	0.179
WUR (0–100)	35.42 ± 17.14	34.50 ± 13.73	+0.92 [−12.95; 14.78]	0.042	0.887

## Data Availability

The data that support the findings of this study are available from the corresponding author upon reasonable request.

## Supplementary Materials

The following supporting information can be downloaded at: https://www.mdpi.com/article/10.3390/nu18121839/s1, Table S1: Per-endpoint distributional checks, paired analyses, sensitivity analyses, and effect-size variants.; Table S2: Outcome values by study phase. Values are presented as mean ± standard deviation for phase 1 and phase 2 of the crossover trial.

## Abbreviations

The following abbreviations are used in this manuscript:

5-LO	5-lipoxygenas
ABBA	3-acetyl-β-boswellic acid
AKBA	3-acetyl-11-keto-β-boswellic acid
BAI	Beck Anxiety Inventory
BDI-II	Beck Depression Inventory II
CPM	conditioned pain modulation
CPT	cold pain tolerance
CRP	C-reactive protein
DFNS	German Research Network on Neuropathic Pain
IL-6	interleukin 6
IκB	inhibitor of kappa B
KBBA	11-keto-β-boswellic acid
mPGES-1	microsomal prostaglandin E_2_ synthase-1
NF-κB	nuclear factor kappa B
NRS	numeric rating scale
PPT	pressure pain threshold
PSQI	Pittsburgh Sleep Quality Index
TRPV1	transient receptor potential vanilloid 1
TSA-II	Thermal Sensory Analyzer II
VAS	Visual Analogue Scale
WHO-5	WHO-5 Well-Being Index
WUR	wind-up ratio
