# Supplementary material for: Effect of Boswellia serrata on Pain Intensity, Central and Peripheral Sensitization, and Pain Modulation in Healthy Volunteers—A Randomized, Double-Blind, Placebo-Controlled, Cross-Over Pilot Trial
Source: Nutrients. 2026 Jun 6;18(12):1839. doi: 10.3390/nu18121839 (PMC13304964; doi:10.3390/nu18121839)
Supplement: Supplementary file 1 [file nutrients-18-01839-s001.zip › nutrients-4270841-supplementary Table S1.pdf]

Supplementary Table S1. Per-endpoint distributional checks, paired analyses, sensitivity analyses, and effect-size variants.

All inferential analyses are paired t-tests on within-subject differences (n = 12 healthy volunteers, crossover design). The Shapiro-Wilk test is computed on the within-subject differences, which is the relevant distributional assumption for the paired t-test. The Wilcoxon signed-rank test is reported as a non-parametric sensitivity analysis. Effect-size variants follow Lakens (2013, doi:10.3389/fpsyg.2013.00863).

Endpoint	n	Boswellia Mean ± SD	Placebo Mean ± SD	Mean diff (B – P)	SD diff	95 % CI of mean diff	Shapiro-Wilk W (diff)	Shapiro-Wilk p (diff)	Paired t	Paired t p	Wilcoxon p (sensitivity)	r (within-subject)	d_z	d_{av}	d_{rm}
VAS (0-100)	12	43.00 ± 21.02	46.67 ± 16.59	-3.67	20.01	[-16.38; 9.05]	0.827	0.02	-0.63	0.54	0.95	0.45	-0.18	-0.19	-0.19
HPT (°C)	12	36.84 ± 2.17	37.59 ± 2.36	-0.75	3.42	[-2.92; 1.43]	0.952	0.66	-0.76	0.47	0.46	-0.14	-0.22	-0.33	-0.33
HDT (°C)	12	34.77 ± 0.64	34.79 ± 0.74	-0.02	0.94	[-0.62; 0.57]	0.870	0.07	-0.09	0.93	0.62	0.08	-0.03	-0.04	-0.04
Hyperalgesia (0-100)	12	20.17 ± 12.66	22.83 ± 15.22	-2.67	8.81	[-8.27; 2.93]	0.945	0.57	-1.05	0.32	0.50	0.82	-0.30	-0.19	-0.18
WUR (NRS)	12	35.42 ± 17.14	34.50 ± 13.73	0.92	21.82	[-12.95; 14.78]	0.959	0.77	0.15	0.89	0.93	0.01	0.04	0.06	0.06
Distance of allodynia (mm)	12	35.26 ± 9.56	34.53 ± 8.22	0.73	13.55	[-7.88; 9.34]	0.905	0.18	0.19	0.86	0.75	-0.16	0.05	0.08	0.08
CPM (%)	12	107.21 ± 19.46	113.73 ± 13.81	-6.52	12.93	[-14.74; 1.69]	0.973	0.94	-1.75	0.11	0.13	0.75	-0.50	-0.39	-0.36
CPT (s)	12	18.13 ± 8.61	18.99 ± 9.36	-0.87	12.04	[-8.52; 6.78]	0.975	0.96	-0.25	0.81	0.87	0.10	-0.07	-0.10	-0.10
BAI	12	3.58 ± 5.02	4.92 ± 6.86	-1.33	2.81	[-3.12; 0.45]	0.870	0.06	-1.65	0.13	0.20	0.93	-0.48	-0.22	-0.17
WHO-5	12	17.67 ± 3.03	16.67 ± 3.92	1.00	2.41	[-0.53; 2.53]	0.936	0.45	1.44	0.18	0.21	0.79	0.41	0.29	0.27
PSQI	12	3.67 ± 2.02	4.33 ± 1.87	-0.67	1.97	[-1.92; 0.58]	0.955	0.71	-1.17	0.27	0.33	0.49	-0.34	-0.34	-0.34
BDI	12	4.33 ± 5.02	4.08 ± 5.16	0.25	3.93	[-2.25; 2.75]	0.970	0.91	0.22	0.83	0.80	0.70	0.06	0.05	0.05

Abbreviations and effect-size definitions: *B, Boswellia serrata; P, placebo; CI, confidence interval; r, within-subject Pearson correlation between Boswellia and placebo measurements; d_z, Cohen's d standardised by the SD of the within-subject differences; d_{av}, standardised by the mean of the two condition SDs; d_{rm}, Cohen's d corrected for the correlation between repeated measures (recommended for between-subjects sample-size planning of follow-up trials). VAS, Visual Analogue Scale; HPT, heat pain threshold; HDT, heat detection threshold; WUR, wind-up ratio; CPM, conditioned pain modulation; CPT, cold pain tolerance; BAI, Beck Anxiety Inventory; WHO-5, WHO-5 Well-Being Index; PSQI, Pittsburgh Sleep Quality Index; BDI, Beck Depression Inventory.*
