# Supplementary material for: Effect of Boswellia serrata on Pain Intensity, Central and Peripheral Sensitization, and Pain Modulation in Healthy Volunteers—A Randomized, Double-Blind, Placebo-Controlled, Cross-Over Pilot Trial
Source: Nutrients. 2026 Jun 6;18(12):1839. doi: 10.3390/nu18121839 (PMC13304964; doi:10.3390/nu18121839)
Supplement: Supplementary file 1 [file nutrients-18-01839-s001.zip › nutrients-4270841-supplementary TableS2.pdf]

Supplementary Table S2. Outcome values by study phase. Values are presented as mean ± standard deviation for phase 1 and phase 2 of the crossover trial.

Parameter	Phase 1	Phase 2
BAI	4.58 ± 3.85	3.92 ± 7.03
BDI	4.75 ± 6.31	3.67 ± 3.37
CPM (%)	112.37 ± 16.1	108.56 ± 18.0
CPT (s)	18.53 ± 8.99	18.58 ± 9.02
Distance of allodynia (mm)	36.61 ± 10.63	33.17 ± 6.32
HDT (°C)	34.79 ± 0.56	34.78 ± 0.80
HPT (°C)	37.3 ± 2.29	37.13 ± 2.31
Hyperalgesia (0-100)	20.2 ± 12.7	22.0 ± 12.42
PSQI	4.58 ± 2.23	3.42 ± 1.44
VAS (0-100)	40.17 ± 21.45	49.5 ± 14.72
WHO 5	17.17 ± 3.49	17.17 ± 3.59
WUR (0-100)	30.00 ± 18.36	39.92 ± 9.59
